# The Effect of 8-Week Protein Supplementation with a Simple Exercise Program on Body Composition, Muscle Strength, and Amino Acid OMICS among Healthy Sedentary Indians: A Randomized, Double-Blind, Placebo-Controlled Trial

**DOI:** 10.1155/2024/5582234

**Published:** 2024-09-03

**Authors:** Sucharita Sambashivaiah, Madhavi Marathe, Rohini Bhadra, Shinjini Bhattacharya, Sumithra Selvam

**Affiliations:** ^1^ Department of Physiology St Johns' Medical College, Bengaluru, India; ^2^ Health Care Nutrition Science & Medical Affairs Nutricia International Pvt Ltd., Mumbai, India; ^3^ Division of Clinical Physiology Department of Physiology St John's Medical College St John's Research Institute, Bengaluru, India; ^4^ Division of Epidemiology and Biostatistics St John's Research Institute, Bengaluru, India

## Abstract

Dietary protein plays a crucial role in the modulation of several physiological processes to sustain health and well-being. There is robust evidence of enhanced muscle protein synthesis, improved physical fitness, body composition, and performance contributed by protein supplementation combined with exercise among trained individuals or athletes. Evidence of the efficacy of such intervention on healthy adults having a sedentary lifestyle is limited. The objective of this study was to evaluate the impact of 12 g of additional protein in the form of a protein supplement compared to a placebo combined with a simple exercise program on plasma amino acid level, body composition, and muscle strength among healthy Indian adults having a relatively sedentary lifestyle. This double-blinded, randomized controlled trial was conducted on sedentary healthy adults 20–45 years of age, with a body mass index (BMI) between 18.5 and 27.9 kg/m^2^. Eighty-two participants were randomized into either the protein (intervention) or placebo (control) group. The exercise regime was the same for both groups. Out of 82 randomized participants, 58 completed the intervention. Blood tests were conducted for the amino acid OMICS measurement followed by dual-energy X-ray absorptiometry (DXA) for body composition and isokinetic dynamometry for muscle strength. A significant improvement was observed in the lean mass (kg) and appendicular muscle mass (AMM) adjusted for weight in the intervention group compared to the control group (*p* < 0.05). The muscle strength and contractile quality were comparable in the 2 groups. Plasma BCAA showed a significant negative association with body fat % (*r* = −0.43, *p* < 0.05 for the intervention group and *r* = −0.33, *p* = 0.07 for the control group) and a positive association with lean body mass % (*r* = 0.56, *p* < 0.01 in the intervention group vs *r* = 0.29, *p* = 0.10 in the control group) in the intervention group compared to control. In conclusion, this study highlighted the value of incorporating a lifestyle intervention including protein supplementation with simple exercises to optimize body composition in sedentary healthy individuals. This trial is registered with CTRI/2018/12/016777.

## 1. Introduction

Dietary protein plays a crucial role in the modulation of several physiological processes to sustain health and well-being [1]. There is a robust amount of evidence on enhanced muscle protein synthesis contributed by protein supplementation when combined with exercise [2]. Adequate protein intake aids in muscle building and maintenance, strengthens the immune system, and may improve bone health [1]. Protein supplementation when combined with exercise has been shown to improve physical fitness and optimize body composition and performance among athletes [3–5]. Similar benefits have also been reported among older adults and patients with chronic diseases [6–8]. Evidence on the efficacy of such intervention among sedentary healthy adults is limited. However, these interventions, if implemented during the early phase of adulthood, may lay the foundation for establishing a healthy lifestyle thereby improving the overall health and minimizing the risk of developing chronic diseases in the future.

Indians have been known to have inadequate intake of protein. As per the National Institute of Nutrition (NIN) and Indian Council of Medical Research (ICMR) report, although the protein-energy ratio of the diet of Indians was as per the recommendation of 10.4% for males and 11.1% for females, the quality of protein was found to be consistently poor across different regions in India. The probability of protein deficiency among Indians based on these data was approximately 36% for the rural population and 44% for the urban population [9]. Furthermore, with an increase in urban lifestyle, the physical activity among the population is gradually decreasing. Recent evidence has highlighted the increased proportion of physical inactivity among the Indian population [10, 11]. Physical inactivity may aggravate muscle deconditioning and lead to decreased muscle mass and strength along with the accumulation of body fat both in subcutaneous and ectopic regions [12]. It has been postulated that the onset of muscle loss may begin as early as the age of 30 years with around 3–8% of tentative muscle loss occurring each decade post 30 years [13]. Notably, the onset of sarcopenia can take place as early as the 4^th^ decade of life [14]. These typical body composition alterations can be aggravated with age resulting in a predisposition to developing chronic diseases [15]. Indians, in general, are known to possess poor muscle mass and function compared to the Western population [16, 17]. Simultaneously, there is a higher propensity to accumulate fat in the intramyocellular region in this population as demonstrated by Sucharita et al. which is postulated to be a major contributor to the widely prevalent metabolic syndrome among this population [16]. Therefore, it is imperative to focus on this population to achieve a higher peak muscle mass and function through early interventions.

The role of increased amino acid in the regulation of post exercise muscle protein synthesis is complex. The introduction of dietary protein-derived amino acids into circulation during the postprandial period is crucial for muscle protein synthesis. A previous study conducted by us demonstrated an increased circulating amino acid level post consumption of a protein supplement among Indian adults [18]. However, this study observed only the acute effect of the protein supplement. It is also important to understand the role of prolonged protein supplementation with a simple exercise regime on the amino acid levels in blood and subsequently the body composition and muscle strength, as this has never been explored among sedentary Indians. The objective of this study was, therefore, to evaluate the impact of a protein supplement compared to a placebo combined with a simple exercise program on amino acid OMICS, change in body composition (lean mass and fat mass), and muscle strength among healthy Indian adults having a relatively sedentary lifestyle.

## 2. Materials and Methods

### 2.1. Participants

This double-blind, randomized controlled trial included sedentary healthy volunteers (both male and female) 20–45 years of age, with a BMI of 18.5–27.9 kg/m^2^ and with an ability to exercise. The participants were screened and recruited from in and around St. John's Medical College and Hospital, Bengaluru, India. Participants were excluded if they had a prior history of any chronic diseases (including but not restricted to diabetes mellitus, prediabetes, hypertension, ischemic heart disease, and renal failure) or any infections (such as tuberculosis, typhoid, and dengue) in the last 3 months or any form of anaemia, acute weight loss (>2 kg in the last 6 months), cancer, lactose intolerance, alcohol intake greater than 1-2 standard servings, any form of neuropathy or muscular dystrophy, joint injury or surgery, pregnancy, high physical activity, food allergy, consumption of any protein or nutritional supplement, or high grip strength (>40 kg for males and >35 kg for females). Eligible participants as per the inclusion and exclusion criteria subsequently underwent tests for fasting blood sugar, glycated haemoglobin (HbA1c), lipid profile, urea, creatinine, and haemoglobin to confirm their health status before being enrolled for the study. The study protocol was approved by the Institutional Ethics Committee (IEC), St John's National Academy of Health Sciences, Bangalore, and registered with the clinical trials registry-India (IEC ref no. 219/2018; CTRI registration no. CTRI/2018/12/016777). Written informed consent was obtained from all participants.

**Figure 1:**
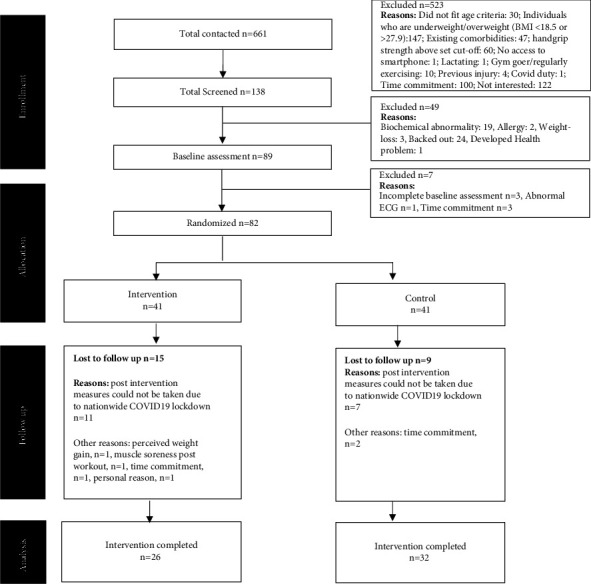
CONSORT flow diagram of the study. BMI: body mass index; ECG: electrocardiogram.

**Figure 2:**
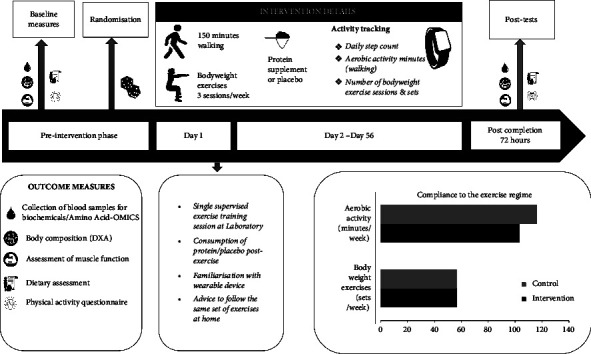
Study flowchart and average compliance.

Out of 661 individuals contacted for this study, 523 individuals had to be excluded for various reasons as described in detail in the CONSORT flowchart illustrated in Figure 1. A total of 138 participants underwent blood screening for haemoglobin, lipid profile, fasting glucose, HbA1c, urea, and creatinine to determine their health status and rule out any undiagnosed metabolic problems as required by the study protocol. Excluding the 49 participants who did not qualify for the study due to abnormality in their blood parameters or due to health issues, and who backed out postscreening, baseline measurements were carried out for 89 participants. Finally, 82 participants were randomized into either intervention or control arms (41 in each arm). Out of 82 participants, the 8-week intervention was completed by 58 participants (26 in the intervention arm and 32 in the control arm). There was a loss to follow-up for 6 participants due to various personal reasons which did not allow them to continue with the study, but post intervention measures of 18 participants who had completed the study could not be taken due to the COVID-19 pandemic and ensuing nationwide lockdown. The experimental protocol followed for each participant has been represented in Figure 2.

### 2.2. Sample Size

The required number of study participants was 26 in each group to detect a difference of 4.5 kg in the hand grip strength between the intervention and control groups [19] with a SD of ∼4 kg, 5% level of significance, and 90% power. Expecting a loss of follow-up of 10%, the sample size of 30 was considered in each group.

### 2.3. Outcome Measures

The following assessments were carried out at the baseline and post intervention for all the enrolled participants.

### 2.4. Body Composition

Anthropometric measurements of height, weight, and body circumferences (midarm, waist, and hip) were carried out for all participants using standard protocols. BMI and waist-hip ratio were derived from the above mentioned measurements. The body composition of the participants including body fat and lean mass was measured using dual-energy X-ray absorptiometry (DXA, Lunar Prodigy Advanced PA + 301969 whole body scanner with software version 12.30, GE Medical Systems, USA). DXA scans were performed with the participants wearing light clothing and without any metal objects in their possession. The participants were asked to lie down under the DXA scanner for approximately 5 minutes, the time taken to complete the scan. The mass of lean soft tissue and fat for the whole body and specific regions were obtained from the DXA scans. The appendicular muscle mass (AMM) was obtained by the sum of the lean tissue of the 4 limbs and the AMM corrected for height and weight were derived from the direct measurement of the muscle mass of the four limbs as obtained from the DXA scans. The AMM corrected for height was derived from the formula AMM/(height)^2^ and the AMM corrected for weight was derived from the formula AMM/weight.

### 2.5. Muscle Strength and Contractile Quality

An isokinetic dynamometer (Kin Com AP1, Chattanooga Group, Tennessee, USA) was used to measure the skeletal muscle strength of the quadriceps of the right leg. Each participant was checked for any history of injury and dominance of legs before the muscle strength evaluation. Since none of the participants reported any preference for the leg, the right leg was used for the measurement. Before the initiation of the test, a warm-up session was performed for all participants where they were requested to carry out a 5-minute indoor brisk walk. Postwarm-up participants were told to sit erect in the dynamometer with stabilization straps across the hip and chest to avoid the usage of body forces during the experiment. The lateral condyle of the knee served as the dynamometer's axis of rotation, and the distal support pad was positioned close to the malleoli. The distance in the lever arm, between the force-length application point and the dynamometer's centre of rotation, was noted. The lever arm was locked at a position that was 30 degrees of knee joint extension from the flexed (90 degree) position where the maximum isometric torque (0°/s) was measured. Three angular velocities 60, 120, and 180°/s were used to measure the knee extensor's peak isokinetic strength. Between each contraction, the subjects were given a 1-2 minute break, and there was a 2-3 minute break between each velocity. Three trials were recorded for each test. For the analysis, the best of three trials at each velocity was chosen.

For the contractile quality estimate, the AMM was taken into account. The peak torque (Nm) at each angular velocity was divided by the AMM (Nm/kg) to determine the muscle's contractile quality.

Muscle function of the upper limb was evaluated by a load cell (Model TR 12, IPA, Bangalore, India). Maximal voluntary contraction (MVC) was recorded 3 times, with constant encouragement and adequate intervals between each measurement. The best of the 3 trials was used in the analysis. Following the MVC, the rate of decline of the sustained MVC was also recorded, up to when 50% of the maximal value was reached and this rate (kg/s) was taken as an index of static endurance.

### 2.6. Targeted Amino Acid Analysis

The levels of 11 AAs were assessed in the plasma samples. The amino acids assessed were as follows: lysine, methionine, leucine, isoleucine, phenylalanine, threonine, valine, proline, serine, glycine, and tryptophan. The key amino acids which could potentially play a role in muscle protein synthesis have been included. The amino acid extraction and analysis were performed as detailed earlier [18]. In brief, samples were deproteinized and derivatized to their ethoxycarbonyl ethyl esters (ECEEs) and analysed by liquid chromatography-tandem mass spectrometry (LC-MS/MS, 6460 Triple Quadrupole, Agilent Technologies, CA, USA). The total plasma BCAA (valine, leucine, and isoleucine) was calculated by adding the quantities of the 3 BCAA.

### 2.7. Dietary Assessment

A validated food frequency questionnaire (FFQ) was used for assessment of the dietary intake. The FFQ used in the study contains a list of food items belonging to different food groups taken from a food database created over a period of several years at the Division of Nutrition, St John's Medical College [20]. The FFQ had four frequency categories, i.e., daily, weekly, monthly, and quarterly and standard portion size measures. The FFQ questionnaire was administered by a trained nutritionist at baseline and end of the study. Analysis of the nutrient composition of the food item was calculated using standard food conversion tables for ingredients [20]. Both the macro- and micronutrient compositions of the diet were calculated.

### 2.8. Physical Activity Level

The physical activity level (PAL) of the participants was assessed using a validated questionnaire containing activities under various domains such as profession, sleep, exercise/sports, hobbies, household work, sedentary activities, and activities of daily living. The frequency and time spent in each activity along with the MET value were used to calculate the PAL [21].

### 2.9. Intervention

Following screening and baseline measurements, the participants were randomly allocated into either the protein (intervention) or the placebo arm (control). Both groups were assigned and trained for low-to-moderate intensity calisthenic exercise and aerobic exercise in addition to the supplement. A computer-generated random number list was prepared by the statistician. Randomization was performed by a researcher not involved in the study. The study personnel did not have access to the randomization list. Both the protein and placebo powders were packed in identical sachets which were coded. The products were identical to each other in colour, texture, and packaging to make them indistinguishable from one another. All study personnel, participants, and the statistician were blinded to the product allocation. In both groups, participants were asked to ingest the supplement once daily preferably during midday or evening between meals. They were asked not to consume the supplement along with or as a replacement of their meals.

### 2.10. Test Protein Supplement and Placebo

Each test protein supplement consisting of skimmed milk powder and soy protein isolates (Protinex, Nutricia International Pvt. Ltd, India) and placebo (maltodextrin-based powder) weighed 35 g each. The protein supplement was fortified with micronutrients, while the placebo contained only carbohydrates and was devoid of protein and micronutrients. A detailed composition of the test protein supplement and the placebo has been presented in Supplementary Table 1. Both the test protein and placebo supplements were prepared by adding the product to 200 ml of lukewarm toned milk. All participants were asked to consume the entire supplement within 10 min of preparation. When dissolved in milk, the test protein supplement provided 244 kcal and 18 g of protein (12 g from the supplement and 6 g from toned milk), while the placebo provided 252 kcal and 6 g of protein (from toned milk). The participants were advised to consume the supplement after the exercise regime. Adherence to the supplement was maintained by each participant in a consumption log throughout the intervention period provided by the study personnel.

### 2.11. Exercise Protocol

In addition to the protein supplement, all participants were instructed to perform an exercise program suitable to be performed in a home environment with minimal external resources. The exercise regime included aerobic exercise (150 minutes of walking) and 3 sessions of low-to-moderate intensity calisthenic exercise per week for the entire duration of the intervention (8 weeks). The participants were instructed and encouraged to walk for at least 30 minutes at each walking session. The first session of the calisthenic exercise was a supervised training session carried out by a qualified physiotherapist post which the participants were provided with instructional videos and a manual to be followed at home. The calisthenic regime consisted of squats, hip bridges, forward lunges, step-ups, push-ups, plank, jumping jacks, and calf raises. Participants were asked to complete three sets of each exercise with 12 repetitions in each set. The calisthenic regime was preceded and followed by warm-up and cool-down stretches to prevent injuries. The simple exercise routine was asked to be completed by the participants whenever it best suited their daily schedule.

Each participant received a wearable activity tracker (Garmin Vivosmart 3) to track their activity during the intervention phase in order to ensure exercise compliance. The participants were asked to wear the fitness tracker every day for the entire 8-week duration. They were asked to record every session of walking and calisthenics in the tracker. In addition, the tracker would continuously monitor the step count of the participants throughout the day. The compliance to the exercise was monitored by the study personnel and the data obtained from the tracker have been presented in Supplementary Table 2. Daily reminders were sent to the participants mentioning the amount of walking and calisthenics pending for the week as observed from the tracker. The participants were advised not to engage in any new exercise regime over and above the prescribed exercises for the course of the intervention period.

### 2.12. Statistical Methods

The assumption of normality was assessed. Mean with standard deviation and median with 25th and 75th percentiles were reported for the study variables as per the assumption of normality. An independent *t*-test or Mann–Whitney *U* test was used to compare the study outcomes between the intervention and control groups. Percentage change from baseline was computed and compared between the intervention and control groups using the Mann-Whitney *U* test. Percent fat, percent lean mass, AMM, and AMM(adjusted for weight) were compared between intervention and control groups using multiple linear regression adjusted for covariates. Spearman's rank correlation was used to assess the relationship of body fat and lean mass (in percentage) with plasma branched-chain amino acid. A *p* value of less than 5% was considered statistically significant. All the analysis was performed using SPSS version 25.0.

## 3. Results

Among the total 82 participants randomized in this study, 41 participants were enrolled in each of the intervention and control groups. A schematic representation of the study participants' recruitment and allocation is presented in the CONSORT diagram in Figure 1. A total of 26 and 32 participants, respectively, in the intervention and control groups completed the follow-up post intervention measures and were therefore considered in the final analyses. Eleven and seven participants in the intervention and control groups, respectively, were lost to follow-up due to the COVID-19 pandemic and ensuing lockdown. As COVID-19 was the primary cause of dropouts and the number of participants completing the trial satisfied the required sample size, an analysis was conducted in accordance with the per-protocol approach.

The mean age, PAL, anthropometric measurements (height, weight, midarm circumference, and waist-hip ratio), body composition, muscle strength, and contractile quality were comparable at baseline and represented in Supplementary Table 3. The average consumption of energy and macronutrients (carbohydrate, protein, and fat) between the 2 groups at baseline has been represented in Supplementary Table 3 and was comparable between the 2 groups. The intervention group had a mean dietary intake of 1994 kcal, 68 g fat, 281 g carbohydrate, and 75 g protein (1.14 g/kg body weight/day, up from 1 g/kg body weight/day), while the control group had a mean dietary intake of 2006 kcal, 65 g fat, 290 g carbohydrate, and 62 g protein (1 g/kg body weight/day maintained throughout the study) at the end of the intervention period with no significant difference in the energy and macronutrient intake except protein (*p* = 0.03) between the groups. The percentage change in protein consumption was also significantly different in the 2 groups (*p* = 0.04) with the intervention group showing a 10.7% change in protein consumption and the control group showing only a 1.4% change. The comparison of the pre-intervention, post intervention, and percentage change of anthropometry, body composition, muscle strength, and contractile quality between the participants who completed the trial has been presented in Table 1. Post intervention lean mass (kg) and AMM adjusted for weight were significantly greater in the intervention group compared to the control group (lean in kg was 43.6 vs. 39.0; AMM (weight) was 0.42 vs. 0.39; *p* < 0.05) (Table 1). The percent lean mass was also noted to be higher in the intervention group compared to controls. A decrease in fat mass and fat percentage was observed in the intervention group compared to the control group, although the differences were not statistically significant. A significant decline in the % change in the fat mass was observed in the intervention group (*p* = 0.04). The muscle strength and contractile quality were comparable between the study groups.

Post intervention percentage fat and lean, AMM, and AMM adjusted for weight were compared between intervention and control groups using multiple linear regression adjusted for covariates such as age, BMI, and physical activity and are represented in Table 2. Percent fat was significantly lower (*β* = −4.51; *p* = 0.02) among participants in the intervention group compared to controls. Similarly, AMM in kg (*β* = 2.16; *p* = 0.04) and AMM adjusted for weight (*β* = 0.034; *p* = 0.03) were significantly higher in the intervention group compared to the control group. Although statistically not significant, participants in the intervention group showed greater improvement in percent lean mass (*β* = 3.32; *p* = 0.07) compared to the control group.

**Figure 3:**
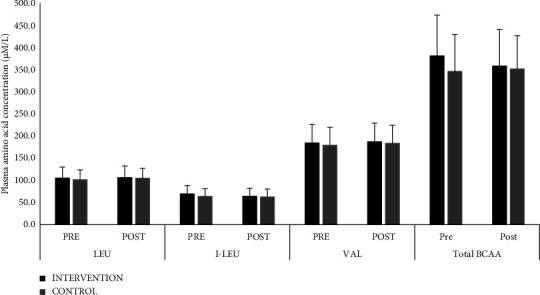
Comparison of plasma concentration of total and individual branched-chain amino acids between groups, represented as mean with SD bars. LEU: leucine; I-LUE: isoleucine; VAL: valine; BCAA: branched-chain amino acid.

**Figure 4:**
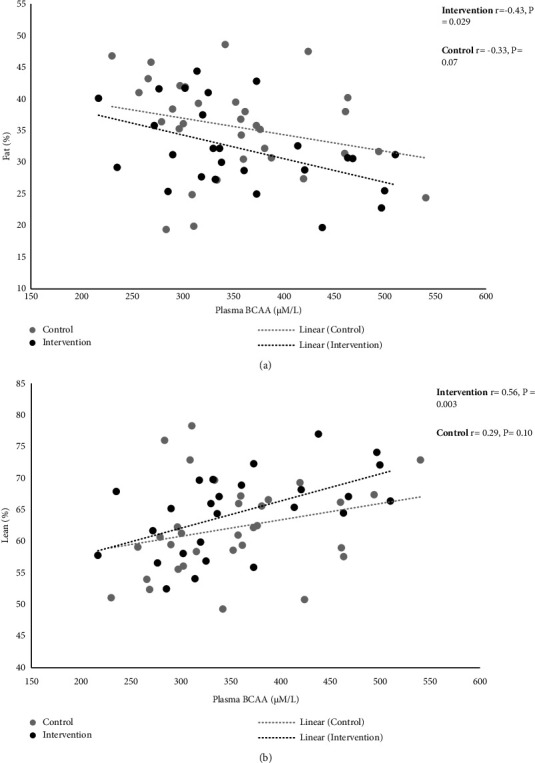
Association of body fat (a) and lean mass (b) with plasma branched-chain amino acid. Plasma BCAA: total plasma branched-chain amino acid (i.e., leucine, isoleucine, and valine) concentration.

The comparison of all analysed amino acids between the 2 groups was comparable and has been represented in Supplementary Figure 1. The comparison of BCAA concentration between the intervention and control groups of pre and post intervention is represented in Figure 3 and was noted to be comparable. In addition, the correlation between post intervention plasma branched-chain amino acid levels and body fat and lean percentage within each study group has been illustrated in Figure 4. Plasma BCAA showed a significant negative correlation with body fat % (*r* = −0.43, *p* < 0.05 for the intervention group and *r* = −0.33, *p* = 0.07 for the control group) and a positive correlation with body lean mass % (*r* = 0.56, *p* < 0.01 in the intervention group vs *r* = 0.29, *p* = 0.10 in the control group). The correlation between percentage change in plasma BCAA and percentage change in lean percent showed a significant positive correlation in the intervention group only (*r* = 0.410; *p* = 0.038); however, no correlation was observed with percentage change in fat percent in either group.

## 4. Discussion

The study demonstrated a significant improvement in lean mass (kg), appendicular muscle mass adjusted for body weight, and reduction in fat mass following daily protein supplementation with a structured simple low-to-moderate intensity calisthenic exercise and walking regime for 8 weeks among sedentary healthy Indian adults. We further explored the association of plasma essential amino acids especially BCAA with changes in body composition. The circulating plasma amino acid level including BCAA was similar in both the intervention and control arm at the end of the study, despite a strong and significant association of both lean mass and fat mass with BCAA observed in the intervention arm only (and not in the control arm). The findings of the present study provide valuable insights into the role of lifestyle modification programs (consisting of protein supplementation and simple exercise programs) among sedentary Indian adults. The momentum towards the lifestyle program is targeted to improve disease state or prevent it. However, there are lack of studies focusing on apparently healthy individuals and lifestyle programs. It is important to focus on healthy individuals as the shift from healthy to a disease state especially a lifestyle-based disease state occurs much quicker. Early sensitization and adaptation of targeted lifestyle intervention programs are essential. The present study is an attempt in this direction.

The improvement observed in body composition, especially lean mass highlights the positive role of the intervention to preserve lean muscle. Our findings are consistent with the recent studies investigating the role of protein supplementation with resistance exercise among healthy adults. Oh et al. investigated the effect of leucine-rich protein supplements containing 20 g of protein with 2 g of leucine for 12 weeks with daily resistance exercise and reported improvement in body composition as evidenced by a significant decrease in body fat and improvement in lean body mass among healthy adults [22]. The study included slightly older adults (i.e., age >50 years) with participants performing resistance exercises using TheraBand with or without a leucine-rich protein supplement for 12 weeks [22]. In our study, we demonstrated similar improvement in body composition using a simple calisthenic exercise protocol in combination with protein supplementation among healthy adults in a shorter duration than the earlier study. Another study investigating the effect of 8-week mung bean supplementation with 18 g protein among underactive vegetarian adults found improvement in percentage change in muscle strength indices in the protein group (strength for grip, flexor, and extensor strength), but change in lean body mass was not significantly different between the groups [23]. The authors speculated that the lack of a few essential amino acids in mung beans could have failed to generate sufficient anabolic stimulus and may have contributed to such results. We included a high-quality protein mixed with milk that is rich in essential amino acids and has a protein digestibility-corrected amino acid score (PDCAAS) of 1, like pure whey protein powder that could contribute to improvement in lean body mass. We observed improvement in muscle strength in both intervention and control arms but the difference was not statistically significant between the arms. Hulmi et al. demonstrated similar responses with reports of enhanced muscle hypertrophy after consumption of 15 g whey protein immediately following resistance training for 21 weeks and with similar muscle strength between protein and placebo groups [24]. The mechanisms contributing to the gains in mass and strength following protein supplementation among untrained adults remain unclear [25, 26]. The improvement in strength could depend on the training driven by the neural mechanisms i.e., the number of active motor units and/or an increase in their firing frequency. The neural activation leading to improvement in muscle strength might need longer training for the effect to appear [27]. Considering the well-established positive correlation between muscle mass and muscle strength [28–30], the change in the protein arm could, therefore, become significant and more consistent in terms of both muscle mass and muscle strength production after much longer-term training (e.g., 1-2 years) or possibly even faster with the inclusion of already well-trained subjects. These possibilities need further investigation. Contrary to our findings, Hida et al. studied the effect of an 8-week egg protein supplement of 15 g on muscle strength and serum-free amino acid concentrations among healthy female athletes and observed no change in body composition or muscle strength [31]. Similarly, another study investigating the effect of high protein intake (>3 g/kg/day) compared to normal protein intake (habitual) among healthy resistance-trained men found no significant difference in body composition at the end of four months of intervention [32]. Notably, both above mentioned studies were conducted on trained athletes who may represent higher fitness levels, functional capacity, and different body composition at baseline than our study population, Nevertheless, based on available evidence, it seems plausible that the addition of protein supplementation with exercises may favour improving body composition parameters of sedentary healthy adults.

In the current study, the circulating plasma amino acid levels including BCAA were similar in both intervention and control arms at the end of the study. This is contradictory to our previous finding where an acute dose of protein supplement significantly increased circulating amino acid (including BCAA) levels compared to placebo [18] The lack of difference in BCAA in the present study could be attributed to greater utilization of the essential plasma amino acids for muscle protein synthesis as evidenced by the significant improvement in body composition parameters. In a study by Bigard et al. [33], the effect of protein supplementation during seven-stage mountaineering at moderate altitude among highly trained individuals was studied. It was observed that there was a significant decrease in the plasma BCAA levels in the control group at the end of the program, and the high protein intake group attenuated the exercise-induced decrease in BCAA. Duration of training, intensity, and activity status of an individual appear to be important factors contributing to improved efficiency of amino acid utilization [34, 35].

In this study, a stronger and significant association of body composition with BCAA was observed in the intervention arm only (and not in the control arm) despite having similar circulating BCAA levels in both groups. Evidence has shown muscle protein synthesis (MPS) to be primarily triggered by a postprandial rise in circulating essential amino acids (EAA) particularly BCAA due to their ability to independently stimulate MPS [36]. BCAA may serve as an alternative fuel source sparing muscle glycogen during prolonged exercise and may reduce exercise-induced muscle fatigue [37]. This most plausibly explains the improvement in lean mass observed in this study. The mechanisms of action of the protein supplement to reduce body fat are not fully clear at this point, however, increased thermogenesis, stimulation to regulate fat metabolism in adipocytes and myotubes by leucine (i.e., inhibition of fatty acid synthase expression in adipocytes and increasing fatty acid oxidation in myotubes), and regulation of appetite hormones associated with high protein consumption have been suggested to contribute to reduction in body fat [38].

The study has several strengths, including the randomized study design, objective measurements, and appropriate statistical analysis which contribute to the reliability and validity of the findings, enhancing the overall quality of the study. Though the required sample size was achieved, few of the allocated participants were lost to follow-up after completing the whole intervention due to the nationwide COVID-19 lockdown which can be considered as a limitation of the study. The study would have benefited from all participants completing the endpoint follow-up. In addition, evaluating the effect of the intervention on thermogenesis (energy metabolism) and GLP-1 secretion was beyond the scope of the study, and this would have added value to the study and has been listed as one of the limitations of the study.

## 5. Conclusion

In conclusion, this study highlighted the value of incorporating lifestyle intervention in the form of simple exercises along with a protein supplement, to optimize body composition in sedentary healthy individuals. The incorporation of a high-quality protein supplement rich in BCAA might amplify the effectiveness of a simple exercise routine.

## Figures and Tables

**Table 1:** Change in body composition, muscle strength, and muscle quality across groups.

Parameters	Intervention (*n* = 26)			Control (*n* = 32)		
	Preintervention	Postintervention	% change	Preintervention	Postintervention	% change
Weight (kg)	65.9 ± 9.16	66.1 ± 9.2	0.51 (−1.38, 1.42)	62.7 ± 10.1	63.3 ± 10.4	1.36 (−0.65, 2.94)
BMI (kg/m^2^)	23.7 ± 2.27	23.9 ± 2.3	0.20 (−0.94, 1.61)	23.3 ± 2.76	22.9 ± 3.3	0.93 (−1.62, 2.85)
Waist-hip ratio	0.81 ± 0.09	0.78 ± 0.05	−1.32 (−4.35, 0.89)	0.78 ± 0.07	0.76 ± 0.07	−2.03 (−5.13, 0.00)
Fat (kg)	21.2 ± 5.5	20.2 ± 3.9	−1.54^#^ (−5.52, 1.94)	21.0 ± 4.5	21.4 ± 5.0	2.13 (−2.42, 5.45)
Fat (%)	32.5 ± 7.17	32.1 ± 6.6	−0.80 (−5.33, 2.04)	35.5 ± 6.87	35.6 ± 7.5	0.25 (−2.48, 4.12)
Lean mass (kg)	41.9 ± 8.5	43.6 ± 8.4^*∗*^	0.79 (−1.55, 3.05)	38.2 ± 8.8	39.0 ± 8.4	1.82 (−1.32, 3.38)
Lean (%)	63.5 ± 7.38	64.6 ± 6.4	1.06 (−1.85, 3.53)	61.3 ± 8.50	62.1 ± 7.2	0.64 (−2.71, 2.38)
AMMI (kg/m^2^)	9.76 ± 1.46	10.0 ± 1.4	1.64 (−2.62, 3.94)	9.05 ± 1.48	9.25 ± 1.5	2.58 (−0.70, 4.67)
AMM adjusted for weight (kg/kg)	0.40 ± 0.05	0.42 ± 0.05^*∗*^	1.19 (−0.29, 2.95)	0.39 ± 0.05	0.39 ± 0.05	0.45 (−2.32, 3.40)
Maximum voluntary contraction (kg)	24.8 ± 7.01	26.5 ± 7.7	6.25 (0.00, 13.0)	22.6 ± 7.76	23.5 ± 7.9	3.03 (0.00, 8.33)
Endurance (kg/sec)	2.60 (0.93, 5.74)	5.58 (1.92, 9.06)	53.7 (−34.3, 363.2)	2.79 (1.08, 7.23)	4.84 (2.44, 6.69)	7.06 (−37.3, 97.8)
Isometric muscle strength (Nm)	67.5 ± 15.0	66.5 ± 12.7	1.42 (−7.51, 8.43)	58.0 ± 14.4	61.4 ± 19.1	1.25 (−3.33, 13.5)
Isokinetic muscle contraction at 60° (Nm)	53.4 ± 15.8	58.5 ± 16.9	10.9 (−11.7, 30.6)	48.1 ± 11.8	56.7 ± 21.5	20.0 (−4.25, 34.4)
Isokinetic muscle contraction at 120° (Nm)	44.9 ± 14.4	51.3 ± 14.8	20.0 (−11.6, 44.1)	44.6 ± 14.1	50.2 ± 18.5	16.7 (−9.55, 44.5)
Isokinetic muscle contraction at 180° (Nm)	43.5 ± 15.9	47.2 ± 13.3	2.32 (−9.80, 37.0)	39.5 ± 13.5	48.6 ± 24.4	9.41 (0.00, 58.5)
Isometric muscle quality (Nm/kg)	2.57 ± 0.50	2.43 ± 0.53	0.80 (−14.7, 6.70)	2.45 ± 0.54	2.53 ± 0.58	0.29 (−6.89, 14.06)
Isokinetic muscle quality at 60° (Nm/kg)	1.99 ± 0.51	2.23 ± 0.70	11.9 (−8.03, 29.1)	2.05 ± 0.54	2.31 ± 0.61	17.0 (−7.14, 28.1)
Isokinetic muscle quality at 120° (Nm/kg)	1.66 ± 0.43	1.93 ± 0.60	18.3 (−12.8, 42.9)	1.88 ± 0.59	2.06 ± 0.57	14.6 (−13.3, 40.4)
Isokinetic muscle quality at 180° (Nm/kg)	1.61 ± 0.50	1.78 ± 0.55	6.58 (−11.8, 39.7)	1.66 ± 0.51	2.07 ± 1.39	6.04 (−23.8, 51.4)

BMI, body mass index; AMMI, appendicular muscle mass index. Reported as mean ± SD for pre- and postintervention and median with 25^th^ and 75^th^ percentiles for % change from preintervention. ^*∗*^*p* < 0.05 for change in the postintervention values between the 2 groups. ^#^*p* < 0.05 for change in the percentage change from pre to post between the 2 groups.

**Table 2:** Multiple linear regression analysis on the effect of intervention compared to control.

	*β* coefficient	95% confidence interval	*p* value
*Percent fat*			
Intervention vs control			
Unadjusted model	−3.48	−7.26, 0.30	0.07
Adjusted model	−4.51	−8.18, −0.83	0.02

*Lean mass (%)*			
Intervention vs control			
Unadjusted model	2.44	−1.22, 6.11	0.19
Adjusted model	3.32	−0.32, 6.97	0.07

*AMM in kg*			
Intervention vs control			
Unadjusted model	3.35	0.04, 6.66	0.05
Adjusted model	2.16	0.25, 6.67	0.04

*AMM adjusted for weight*			
Intervention vs control			
Unadjusted model	0.03	0.01, 0.05	0.05
Adjusted model	0.034	0.004, 0.064	0.03

Adjusted for age, BMI, and physical activity (total step counts) using multiple linear regression.

## Data Availability

The data used to support the findings of this study are available from the corresponding author upon reasonable request.

## Supplementary Materials

Supplementary Materials The detailed description of the protein supplement and placebo used in the study has been described in Supplementary Table 1. The comparison of the exercise data between the 2 groups provided by the activity tracker used in the study has been presented in Supplementary Table 2. The baseline comparison of body composition, muscle strength, and muscle quality between groups has been presented in Supplementary Table 3. The comparison of individual concentrations of all individual amino acids assessed in this study between the 2 groups has been demonstrated in Supplementary Figure 1.
