# Supplementary material for: The Effect of 8-Week Protein Supplementation with a Simple Exercise Program on Body Composition, Muscle Strength, and Amino Acid OMICS among Healthy Sedentary Indians: A Randomized, Double-Blind, Placebo-Controlled Trial
Source: J Nutr Metab. 2024 Sep 3;2024:5582234. doi: 10.1155/2024/5582234 (PMC11387082; doi:10.1155/2024/5582234)
Supplement: Supplementary Materials — The detailed description of the protein supplement and placebo used in the study has been described in Supplementary Table 1. The comparison of the exercise data between the 2 groups provided by the activity tracker used in the study has been presented in Supplementary Table 2. The baseline comparison of body composition, muscle strength, and muscle quality between groups has been presented in Supplementary Table 3. The comparison of individual concentrations of all individual amino acids assessed in this study between the 2 groups has been demonstrated in Supplementary Figure 1. [file 5582234.f1.docx]

**Supplementary Materials**

**Supplementary Table 1: Detailed composition of the nutrition supplement and placebo**

| **Nutrients** | **UoM** | **Nutrition supplement** | | **Placebo** | |
| --- | --- | --- | --- | --- | --- |
|  |  | **Per 100g** | **Per Serve (35g)** | **Per 100g** | **Per Serve (35g)** |
| **Energy** | **kcal** | **366** | **128** | **388** | **135.8** |
| **Carbohydrate** | **g** | **54** | **18.9** | **95.8** | **33.53** |
| **Sugar** | **g** | **30** | **10.5** | **29.5** | **10.325** |
| **Protein** | **g** | **34** | **11.9** | **1** | **0.35** |
| **Fat** | **g** | **1.5** | **0.525** | **0.1** | **0.035** |
| Other |  |  |  |  |  |
| Calcium | mg | 1300 | 475.0 |  |  |
| Iron | mg | 20 | 7.6 |  |  |
| Phosphorous | mg | 900 | 348.0 |  |  |
| Magnesium | mg | 50 | 22.8 |  |  |
| Zinc | mg | 3 | 1.1 |  |  |
| Iodine | mg | 73 | 29.7 |  |  |
| Copper | mg | 400 | 149.3 |  |  |
| Sodium | mg | 400 | 157.4 |  |  |
| Potassium | mg | 870 | 286.4 |  |  |
| Chloride | mg | 360 | 148.3 |  |  |
| Choline | mg | 107.1 | 37.5 |  |  |
| Vitamin A | mcg ret | 1142.8 | 400 |  |  |
| Vitamin B1 | mg | 2.2 | 0.8 |  |  |
| Vitamin B2 | mg | 3.4 | 1.2 |  |  |
| Vitamins B6 | mg | 1.1 | 0.4 |  |  |
| Vitamins B12 | mcg | 0.9 | 0.315 |  |  |
| Niacin | mg | 35.7 | 12.5 |  |  |
| Pantothenic Acid | mg | 2.2 | 0.8 |  |  |
| Biotin | mcg | 23.7 | 8.3 |  |  |
| Vitamin C | mg | 60 | 21 |  |  |
| Folic Acid | mcg | 119.4 | 41.8 |  |  |
| Vitamin D | mcg | 6.57 | 2.3 |  |  |
| Vitamin E | mg | 12.2 | 4.3 |  |  |

**Supplementary table 2: Comparison of exercise data between groups as obtained from the activity tracker**

|  | **Intervention (n=26)** | **Control (n=32)** |
| --- | --- | --- |
| **Aerobic activity** |  |  |
| Aerobic activity minutes per week | 99.3±37.8 | 111.5 ± 67.5 |
| Energy expenditure from aerobic activity per week (Kcal) | 779.5 ± 388.2 | 661.2 ± 374.9 |
| Heart rate maximum (beats/min) | 120.7 ± 25.6 | 114.8 ± 43.8 |
| Heart rate average (beats/min) | 94.2 ± 18.4 | 89.1 ± 37.4 |
| **Calisthenic exercise** |  |  |
| Total number of sets per week | 50.5 ± 19.8 | 50.2 ± 21.5 |
| Number of sessions per week | 2.10 ± 0.82 | 2.09 ± 0.90 |
| Energy expenditure from calisthenic exercise per week (Kcal) | 295.5 ± 142.9 | 236.4 ± 116.9 |
| Heart rate maximum (beats/min) | 131.7 ± 34.9 | 128.1 ± 30.3 |
| Heart rate average (beats/min) | 98.3 ± 19.9 | 93.4 ± 23.0 |
| **Step count** |  |  |
| Average step counts daily | 8848 ± 2789 | 7965 ± 5680 |

All variables comparable between groups (p>0.05)

**Supplementary Table 3: Baseline comparison of body composition, muscle strength and muscle quality across groups**

|  | **Intervention (n=41)** | **Control (n=41)** |
| --- | --- | --- |
| Age (yr) | 26.8±5.4 | 26.6 ± 5.7 |
| Sex (M/F) (n) | 24/17 | 19/22 |
| PAL | 1.46 ± 0.11 | 1.47 ± 0.11 |
| **Dietary Intake** | | |
| Energy (kcal) | 2092 ± 686 | 2013 ± 631 |
| Protein (g) | 66.5 ± 20.9 | 61.1 ± 19.6 |
| Carbohydrate (g) | 301 ± 97 | 291 ± 101 |
| Fat (g) | 69.3± 28.8 | 65.3 ±18.4 |
| **Anthropometry** | | |
| Height (cm) | 165.6 ± 9.7 | 163.3 ± 8.7 |
| Weight (kg) | 65.7 ± 10.7 | 62.21 ± 10.1 |
| BMI (kg/m^2^) | 23.8 ± 2.5 | 23.2 ± 2.6 |
| MAC (cm) | 27.3 ± 2.6 | 26.9 ± 2.8 |
| Waist - hip ratio | 0.80 ± 0.08 | 0.78 ± 0.06 |
| **Body composition** | |  |
| Fat (%) | 33.6 ± 7.3 | 35.4 ± 6.7 |
| Fat mass (kg) | 21.2 ± 5.5 | 21.0 ± 4.5 |
| Lean (%) | 63.5 ± 7.3 | 61.3 ± 8.5 |
| Lean mass (kg) | 41.9 ± 8.5 | 38.2 ± 8.8 |
| AMMI (kg/m2) | 9.5 ± 1.3 | 8.9 ± 1.5 |
| AMM adjusted for weight (kg/kg) | 0.40 ± 0.05 | 0.38 ±0.05 |
| **Muscle strength** | | |
| Maximum voluntary contraction (kg) | 24.4±7.2 | 23.0±7.7 |
| Time to fatigue (sec) | 6.58 (2.49, 10.8) | 6.15 (1.74, 12.2) |
| Endurance (kg/sec) | 2.14 (0.93, 5.28) | 2.12 (1.04, 5.70) |
| Isometric muscle strength (Nm) | 64.7±14.3 | 59.0±13.6 |
| Isokinetic muscle contraction at 60° (Nm) | 52.2±16.3 | 47.2±11.0 |
| Isokinetic muscle contraction at 120° (Nm) | 44.0±16.1 | 42.7±12.7 |
| Isokinetic muscle contraction at 180° (Nm) | 40.9±14.7 | 38.1±12.1 |
| **Muscle Quality** | | |
| Isometric muscle quality (Nm/kg) | 2.55 ± 0.47 | 2.50 ± 0.50 |
| Isokinetic muscle quality at 60° (Nm/kg) | 1.98 ± 0.51 | 2.03 ± 0.51 |
| Isokinetic muscle quality at 120° (Nm/kg) | 1.65 ± 0.48 | 1.81 ± 0.53 |
| Isokinetic muscle quality at 180° (Nm/kg) | 1.58 ± 0.50 | 1.61 ± 0.46 |

All variables comparable between groups (p>0.05)

**Supplementary fig 1 Comparison of plasma concentrations of all individual amino acids between groups**
